# Genetic and Environmental Determinants of Spontaneous Preterm Birth: Focus on Progesterone Receptor Gene Variants

**DOI:** 10.3390/ijms262110659

**Published:** 2025-11-01

**Authors:** Mirta Kadivnik, Kristina Kralik, Siniša Šijanović, Jasenka Wagner

**Affiliations:** 1 Clinic of Obstetrics and Gynecology, University Hospital Center Osijek, J. Huttlera 4, 31000 Osijek, Croatia; mirta.kadivnik@kbco.hr (M.K.);; 2 Department of Obstetrics and Gynecology, Faculty of Medicine, J. J. Strossmayer University, J. Huttlera 4, 31000 Osijek, Croatia; 3 Department of Medical Statistics and Informatics, Faculty of Medicine, J. J. Strossmayer University, J. Huttlera 4, 31000 Osijek, Croatia; 4 Department of Medical Biology and Genetics, Faculty of Medicine, J. J. Strossmayer University, J. Huttlera 4, 31000 Osijek, Croatia

**Keywords:** preterm birth, progesterone receptor, polymorphism, single nucleotide, pregnancy complications, gene–environment interaction

## Abstract

Preterm birth (PTB) is a leading cause of neonatal morbidity and mortality worldwide. This study investigated six single-nucleotide polymorphisms (SNPs) in the maternal and fetal progesterone receptor (*PGR*) gene and their association with spontaneous PTB, considering environmental and clinical risk factors. We conducted a case–control study including two groups of pregnant women (term and preterm, 292 in total) and two groups of newborns (term and preterm, 292 in total), and analyzed *PGR* variants (rs1042838, rs1042839, rs10895068, rs4574732, rs653752, and rs1942836) in relation to maternal age, fetal gender, and pregnancy complications such as vaginal bleeding. Results showed that *PGR* SNPs rs1942836 (OR 0.38, CI 95% 0.15–0.98, *p* = 0.03), rs4574732 (OR 2.4, CI 95% 1.01–5.57, *p* = 0.04), and rs653752 (OR 2.27, CI 95% 1.19–4.34, *p* = 0.02) were associated with PTB when considered in the context of clinical factors, highlighting gene–environment interactions. Our findings underscore the importance of integrating genetic and clinical information for a better understanding of PTB risk.

## 1. Introduction

Preterm birth (PTB), defined by the World Health Organization (WHO) as delivery before 37 weeks of gestation [1,2], affects nearly 10% of pregnancies worldwide [3]. Rates vary substantially, from about 7% in East Asia to over 13% in South Asia, with similar prevalence observed in Croatia (6–7%) [4]. PTB contributes to approximately 70% of neonatal deaths and 75% of neonatal morbidity [5].

Many sociodemographic, dietary, medical, environmental, and pregnancy-related risk factors have been shown to increase the risk of spontaneous preterm birth (sPTB) [6,7]. Ideally, the detection of risk factors for sPTB should occur before conception or in the early stages of pregnancy, enabling preventive interventions.

As it is a multifactorial trait, maternal stress, multiple pregnancies, and exposure to toxins during pregnancy could interact with a genetic predisposition and lead to PTB [5,8]. Interactions between genes and environmental factors can make it difficult to clarify and distinguish between the two effects. For example, some maternal genotypes modify the association between maternal cigarette smoking and infant birth weight [9], as well as the presence of bacterial vaginosis in tumor necrosis factor-α2 carriers, predisposed to spontaneous PTB [10]. Not considering environmental factors when performing a case–control study of genetic association and PTB may lead to misinterpretation of the results.

There are four main groups of environmental factors [11]: risk factors from the mother’s general medical history, risk factors from the mother’s obstetric history, risk factors related to the socioeconomic status of the pregnant woman and her family, and risk factors of the current pregnancy. Among them, we should mention both extremes of maternal age, either in adolescent mothers (age under 17 years) or in older mothers (age above 35 years) [7,12], mothers’ smoking habits [13], vaginal bleeding in the first or second trimester, as well as bleeding due to placental abruption or placenta previa [14], fetal gender, and of course, genetic predisposition for sPTB [15,16], including personal [17,18] and family history [19,20] of sPTB.

Overall, maternal and fetal genetic predispositions contribute substantially to PTB risk and interact with clinical factors.

Besides them, one recognized risk factor for PTB is maternal and/or fetal genetic predisposition. It has been confirmed in many epidemiological studies [20,21,22]. Studies suggest that maternal genetic variants contribute about 20.6% to 25% to the heritability of PTB [18,22].

Progesterone (P4) is known as a critical hormone involved with pregnancy maintenance; its absence or relative absence is associated with pregnancy failure, preterm labor, and other poor outcomes. The physiological effects of P4 are mediated by binding to specific nuclear progesterone receptors (nPR). They act by modulating the expression of specific downstream target genes such as the Gap junction Protein Alpha 1 (*GJA1*), the Prostaglandin-Endoperoxide Synthase 2 gene (*PTGS2*), the oxytocin receptor gene (*OXTR*), and the nuclear factor kappa-light-chain-enhancer of activated B-cells subunit (NF-κB2). Two isoforms of PRs are crucial for the influence of P4 on the onset of labor: PR-A and PR-B.

The progesterone receptor gene (*PGR*) codes for progesterone receptors (PRs). The human *PGR* is located on the long arm of chromosome 11 (cytogenetic band 11q21.1), and it consists of eight exons and seven introns [23].

In previous work, we analyzed genetic factors predisposing to PTB in a Croatian population sample [11,24,25]. We selected six single-nucleotide polymorphisms (SNPs) of the *PGR* and found four of them to be associated with the occurrence of PTB in either mothers or newborns.

In this study, we aimed to combine these genotype data with an analysis of environmental risk factors for PTB to elucidate the environmental genetic influence on PTB.

## 2. Results

In the study, in which 151 mothers with PTB and 151 associated prematurely born newborns, and 141 mothers of the control group with 141 associated term-born newborns participated, six *PGR* variants were analyzed after blood collection and genomic DNA isolation: rs10895068, rs1042838, rs1042839, rs1942836, rs4754732, and rs653752.

### 2.1. Characteristics of Participants

Selected epidemiological and demographic characteristics of the 292 mothers in the case and control groups are shown in Table 1. Data was available for all participants. Basic characteristics of newborns, such as gender and birth weight, are also presented. A statistically significant difference was found in gestational age at delivery, the weight of mothers, and newborn birth weight between the two groups of mothers.

Table 2 presents the distribution of key clinical and environmental risk factors between the two groups. As described in the Materials and Methods section, the study group included mothers with spontaneous pre-term delivery (<37 weeks of gestation) and their preterm newborns. In comparison, the control group consisted of mothers with term deliveries (≥37 weeks) and their term newborns. Table 2 presents the distribution of key clinical and environmental risk factors between the two groups. A statistically significant difference between the two groups of subjects was found in the age of the mothers when they were divided into two subgroups: a subgroup of mothers younger than 35 years and a subgroup of mothers older than 35 years. It should also be emphasized that there was a statistically significant difference in the number of patients who bled during pregnancy between the same two groups.

### 2.2. Distribution of Genotypes and Alleles in the Group of Preterm Births in Relation to Characteristics

The studied group of mothers with PTB and the group of corresponding preterm newborns were then divided into two subgroups, depending on whether a certain risk factor for PTB was present or not. In the analysis, risk factors of the newborn’s gender, the mothers’ BMI, mothers’ age, mothers’ smoking habit, previous PTB in personal history, the presence of PTB in the mother’s family history, and mothers’ vaginal bleeding during pregnancy were included. Although newborns’ gender was not identified as a statistically significant independent risk factor when comparing the preterm and term groups (Table 2, *p* = 0.13), it was included in the subgroup analyses because previous studies have shown that male newborns’ gender is associated with a higher risk of spontaneous preterm birth [26,27,28]. Male fetuses are known to exhibit sex-specific differences in inflammatory and hormonal responses, including progesterone and cytokine signaling pathways, which are relevant to the mechanisms investigated in this study.

The distribution of genotypes and alleles of six selected SNPs of *PGR* was analyzed between the subgroups of mothers and the corresponding newborns, which were divided depending on the mentioned clinical factors of the mother and the newborn.

The association between each *PGR* variant and spontaneous preterm birth was analyzed under dominant, recessive, and overdominant inheritance models, as summarized in Table 3, Table 4 and Table 5. To explore potential gene–environment interactions, subgroup analyses were performed within the PTB cohort of mothers and newborns, stratified according to fetal gender (Table 3), maternal age (Table 4), and the presence or absence of vaginal bleeding (Table 5). The observed differences between inheritance models reflect possible variation in how each SNP influences *PGR* expression or function. This approach follows established guidelines for evaluating multiple modes of genetic effect in association studies [29,30]. For instance, coding variants such as rs1042838 and rs1042839 may display dominant effects due to their direct impact on receptor activity, whereas intronic or regulatory SNPs (e.g., rs4574732, rs1942836) may show associations only under specific inheritance assumptions, consistent with their potential role in transcriptional regulation.

In the subgroups of mothers with PTB, no statistically significant difference was found in the distribution and frequency of alleles and genotypes and genetic inheritance models between the two subgroups of subjects in relation to any of the PTB risk factors mentioned (Tables S1–S14, Supplementary Materials).

In the subgroups of preterm-born newborns, a statistically significant difference was found in the distribution and frequency of genotypes between the two subgroups in the case of three risk factors for PTB: newborns’ gender, mothers’ age, and mothers’ vaginal bleeding during pregnancy.

A statistically significant difference in the distribution of rs4754732 genotypes with a higher frequency of the CC genotype was found between subgroups of prematurely born newborns depending on the gender of the newborn, contributing to an almost 2.5-fold higher likelihood of PTB in mothers carrying a male child with the mentioned genotype, according to the recessive genetic inheritance model (TT + CT vs. CC) (OR 2.4, CI 95% 1.01–5.57, *p* = 0.04) (Table 3).

The study and control groups of newborns born to mothers who gave birth to male children were then compared, and no statistically significant difference in the distribution of alleles and genotypes between the above subgroups for any of the six SNPs studied was found (Table S15, Supplementary Materials).

Similarly, a statistically significant difference was found in the distribution of the inheritance model of the rs653752 genotype with a higher frequency of the CC genotype in the group of preterm infants, contributing to a 2.27-fold higher probability of preterm birth in mothers over 35 years of age whose newborn is a carrier of stated genotype, according to the recessive genetic inheritance model (TT + CT vs. CC) (OR 2.27, CI 95% 1.19–4.34, *p* = 0.02) (Table 4).

Subsequently, the groups of term and preterm newborns born to mothers older than 35 years were then compared. No statistically significant difference was found in the distribution of alleles and genotypes for any of the six SNPs of *PGR* (Table S16, Supplementary Materials).

Finally, a statistically significant difference in the distribution of rs1942836 of genotypes was found with a higher frequency of the heterozygous CT genotype in the group of prematurely born newborns whose mothers had no bleeding during pregnancy, which contributed to an approximately threefold lower probability of PTB in the same mothers according to the recessive genetic inheritance model (TT + CT vs. CC) (OR 0.38, CI 95% 0.15–0.98, *p* = 0.03) (Table 5).

Afterwards, the groups of preterm and term newborns born to mothers who had bled during pregnancy were compared, and there was no statistically significant difference in the distribution of alleles and genotypes of any of the six SNPs of *PGR* studied (Table S17, Supplementary Materials).

## 3. Discussion

Despite numerous studies on the mechanisms of its development and on preventive and therapeutic measures that could and should influence its incidence, preterm birth remains one of the main causes of a high percentage of perinatal mortality and morbidity [31]. In addition, the incidence of preterm birth is not decreasing worldwide despite the preventive measures applied [31]. This is partly due to the higher number of medically induced preterm births due to various indications related to other pathological changes associated with pregnancy [32], and partly it may be a consequence of the lowering of the viability threshold of pregnancy and thus the birth of a certain number of newborns born extremely early, in the window of 20 to 24 weeks of gestation.

PTB is considered a major obstetric syndrome because of its multifactorial etiology and overlapping mechanisms [33]. This complexity makes it challenging to identify precise causes or to design effective preventive strategies.

Beyond the spectrum of etiological factors that cause PTB, the genetic predisposition to PTB has increasingly become the focus of research. It has been shown that mothers with a personal or family history of PTB have an increased risk of PTB [18,19]. As one of the most important mechanisms leading to PTB, it is certainly worth highlighting the influence of P4 on the maintenance of pregnancy and possible alterations in its signaling that could lead to PTB. Indeed, it is known that, unlike other mammals, humans do not experience the classic drop in serum P4 levels that leads to, among other things, childbirth. In humans, however, we are familiar with the phenomenon of functional P4 withdrawal (FPW), which is caused by a change in the function or expression of PR to which P4 binds. This process could therefore be one of the triggers for PTB [34]. In line with the previous comments, the investigation of SNPs in the *PGR* and their association with PTB has begun, with the assumption that certain single-nucleotide polymorphisms of the *PGR* are at least partially responsible for the increased odds ratio of PTB.

The study was conducted as a genotype-phenotype association study to determine which *PGR* SNPs are associated with PTB. The aim was also to show whether only the maternal genotype influences the risk for PTB or also the fetal genotype, and whether certain SNPs act independently or within a haplotype, and whether there is a specific interaction of individual SNPs in modulating the risk for sPTB. The results were mentioned in the previous two articles we published [24,25].

In continuation of previous research, we decided to also investigate the association of the six SNPs in *PGR* in combination with risk factors for PTB to this extent. Based on the available literature, this is the first study to investigate the interaction and influence of this combination of six *PGR* SNPs on PTB in both the mother and the associated newborns, as well as possible environmental factors influencing PTB.

The association of six selected SNPs in *PGR* with clinical characteristics of patients and their respective newborns was investigated in a group of mothers with PTB and their respective preterm newborns. Of the risk factors shown to be associated with a higher incidence of PTB, we selected maternal age, gender of the newborn, maternal BMI, maternal tobacco smoke exposure, maternal bleeding during pregnancy, previous preterm birth in the mother, and a history of PTB in the mother’s family.

Maternal age shows a U-shaped risk curve for PTB, and women younger than 17 and older than 35 years have the highest risk [35]. Numerous studies have shown that PTB is related to the age of the pregnant woman. Fuchs et al. have shown that the risk of PTB is 1.5-fold higher in mothers older than 40 years, even after adjustment for covariates [36]. Similar results were also obtained by Lawlor et al. [37]. The increased odds ratio for PTB in older mothers is certainly a consequence of other pregnancy-related complications, such as pre-eclampsia and gestational diabetes, occurring at older ages.

As there were no mothers younger than 17 years in our study, it was possible to divide the group of mothers studied into two groups according to age: those who were older and those who were younger than 35 years. The group of preterm newborns was also divided into two groups according to the age of the mothers. A statistically significant difference was found in the distribution of the homozygous CC genotype of the SNP rs653752 *PGR* between the subgroups of preterm newborns born to older mothers and the subgroup of preterm newborns whose mothers were younger than 35 years. In a recessive inheritance model, this genotype increases the odds ratio for PTB in mothers older than 35 years by 2.27-fold. However, when comparing this genotype between the studied subgroup and the control group of term newborns whose mothers were older than 35 years, no statistically significant difference was found in the distribution of the genotypes of the stated SNP, which is certainly interesting information.

The rs653752 has been less studied in the context of PTB. It is an intronic variant located in the regulatory region of *PGR* and is thought to influence *PGR* expression. To date, five studies have been conducted to investigate the association of the rs653752 SNP of *PGR* with a higher or lower incidence of PTB. Only Ehn et al. found a significant association between the above-mentioned SNP of *PGR* and PTB. Namely, Ehn et al. found the association of the aforementioned SNP in mothers with early PTB and with PTB in general. In our study, there was no statistically significant difference in the frequency of alleles and distribution of genotypes between the groups of prematurely born newborns and newborns born at term who are carriers of this *PGR* SNP.

Thus, advanced maternal age remains an independent contributor to PTB risk, often through associated complications.

Fetal gender is also a risk factor for PTB. A large meta-analysis by Zeitlin et al. showed that the probability of PTB is 1.1 to 1.3 times higher in mothers carrying male infants [26]. Similar results were also shown in the study by Vatten et al. [27]. The mechanisms leading to a higher likelihood of PTB in male infants are not yet fully understood but may involve proinflammatory conditions that are more common in pregnancies with male infants. For example, Challis et al. showed increased expression of Toll-like receptor 4 (TLR-4) in the placenta of pregnancies with male infants [28]. They also found that the response of the trophoblast to these proinflammatory influences could explain, at least in part, the association between PTB and the sex of the male infant. Other explanations for this association include changes in androgen levels in male infants leading to increased estrogen levels and the onset of labor, as well as increased interleukin-1 (IL-1) levels in the amniotic fluid of male preterm infants [38].

In this study, the association of the homozygous CC genotype of the rs4754732 SNP of *PGR* was found in newborns with preterm birth whose mothers carried a male infant in a recessive inheritance model. The odds ratio for PTB increased 2.5-fold. Based on the results obtained, the frequency of alleles and genotypes of the aforementioned SNP with the status of carrying a male was analyzed between the subgroups of preterm and term infants, and no statistically significant difference was found between these two groups.

The rs4754732 is located in the promoter region of *PGR*, and its effect influences the ratio of PR-A and PR-B. So far, its association with PTB has only been highlighted in two studies, so Ehn et al. [39], while Manuck et al. did not [40]. It has also been associated with breast cancer [41]. Considering that one of the possible mechanisms of PTB influenced by the male sex of the newborn is a mechanism involving the metabolism of steroid hormones, and the mechanism that the rs4754732 influences is the influence on the PR ratio; it is possible that their interaction and the influence on the progesterone pathway of pregnancy maintenance together lead to a modulation of the risk of PTB, while each of them alone is not sufficient for this. This is an aspect that should be further investigated.

Overall, male fetal gender appears to increase PTB risk, likely due to proinflammatory and hormonal pathways.

Vaginal bleeding in pregnancy is associated with the occurrence of placental abruption, pre-eclampsia, intrauterine growth retardation, premature rupture of membranes and preterm labor [42]. If bleeding occurs early in pregnancy, the likelihood of severe pre-eclampsia and early PTB is higher. Patients with constant intermenstrual bleeding throughout pregnancy have a higher risk of PTB than patients who only bleed sporadically per trimester.

The mechanism that leads to PTB and thus to bleeding during pregnancy is associated with the formation of thrombin in the decidua. As a result of the increased amount of thrombin, PR expression in the decidua cells is inhibited, which subsequently leads to preterm premature rupture of membranes (PPROM) or placental abruption and consequently to PTB [43].

In brief, vaginal bleeding reflects underlying placental or inflammatory mechanisms that substantially elevate PTB risk.

In contrast to other polymorphisms, the rs1942836 SNP of *PGR* showed a protective effect in newborns. In the overdominant inheritance model, the heterozygous CT genotype of rs1942836 in the newborn contributed to a 2.5-fold lower risk of PTB in the subgroup of mothers who had no hemorrhage and whose newborn was a carrier of the aforementioned polymorphism. When we compared the studied subgroups of mothers who had hemorrhages and the corresponding preterm infants with the subgroups of mothers who had hemorrhages and the corresponding newborns, again, no significant difference was found in the distribution of the genotypes of the SNP in question.

The rs1942836 is a single-nucleotide polymorphism that influences the change in PR expression in favor of PR-B and can therefore indirectly influence the occurrence of PTB. In this case and given the same mechanism of action that it has and bleeding during pregnancy in the mother, it could be a good additional marker for PTB in mothers who have some form of bleeding during pregnancy.

This finding suggests a potential protective role for rs1942836, warranting further research as a possible clinical marker.

What is also interesting about these results is that although certain SNPs were found to be potentially associated markers for PTB with the risk factors like maternal age, gender of the child and bleeding during pregnancy in the mother, given the statistical insignificance in the distribution of genotypes of these same SNPs between the subgroups of subjects and the control group in relation to the same risk factors, the potentially associated markers are not independent risk factors for PTB. Accordingly, the multifactorial and highly heterogeneous etiology of PTB and the interplay of multiple mechanisms that may simultaneously lead to PTB and that may overlap in the selected study groups of mothers and newborns must be reiterated. It is well known that in the interaction between genes and the influence of environmental factors, it is sometimes difficult to separate which influence on a phenomenon has a stronger or independent effect. An example of this is the study by Macones et al. in which bacterial vaginosis influenced the increased odds ratio for PTB in mothers carrying a single nucleotide polymorphism in the TNFα gene [10].

Hence, the identified SNPs may act as modifiers rather than stand-alone risk factors, underscoring the complexity of PTB etiology.

In the available literature, only Luo et al. have investigated the association of certain risk factors for PTB with certain SNPs of the *PGR*, among others [44]. In his study of 78 subjects and 415 controls, Luo investigated the association of three SNPs of *PGR*, the PROGINS variants (rs1042838 and rs1042839) and rs10895068, and included the risk factors maternal age, parity, ethnicity, maternal BMI, maternal bacterial vaginosis, and male sex of the newborn in the analysis. He found an association of the PROGINS variant with PTB only in the group of individuals with a BMI of less than 18.5 kg/m^2^.

This study provides one of the first comprehensive analyses of six *PGR* SNPs in combination with environmental factors, with a well-defined case–control design and stringent exclusion criteria. Strengths include the homogeneous population sample, careful adjustment for clinical variables, and combined evaluation of maternal and fetal genotypes.

However, limitations must be acknowledged: the single-center design and relatively modest sample size may limit generalizability, and the study lacked replication in an independent cohort. Furthermore, only selected SNPs of *PGR* were analyzed, and potential gene–gene or broader environmental interactions were not assessed. Future multi-center studies with larger populations are needed to validate these findings and to explore their translational potential. Also, it is important to say that despite rigorous exclusion criteria, it is not possible to eliminate all potential etiologies of PTB. Future multicenter studies with larger samples and detailed phenotypic data are needed. At the end, further studies with larger cohorts should consider stratifying PTB by gestational sub-categories to identify potential genotype-environment associations specific to extremely or very preterm births.

## 4. Materials and Methods

### 4.1. Study Subjects

This case–control study was conducted between November 2017 and February 2022. at the Clinic of Gynecology and Obstetrics of the Clinical Hospital Center in Osijek and the Department of Medical Biology and Genetics at the Faculty of Medicine in Osijek, Croatia.

The study included 560 participants from the Clinic of Gynecology and Obstetrics, University Hospital Centre Osijek. In accordance with the case–control study design, participants were divided into two groups:

Case group (women with sPTB): 140 mothers and their 140 corresponding preterm newborns.

Inclusion criteria for the sPTB group were delivery before 37 weeks of gestation, spontaneous in onset, not medically induced, and a singleton pregnancy.

Exclusion criteria for the sPTB group:

All known causes of sPTB include conception via assisted reproductive technology, multiple pregnancy, previous cervical surgery, proven lower genital tract infection, kidney disease, gestational diabetes mellitus, and hypertension in pregnancy.

Additionally, all mothers whose newborns were stillborn and whose newborns had congenital anomalies were excluded. At the end, mothers and newborns with confirmed or suspected infection were excluded based on clinical presentation, elevated inflammatory markers, positive vaginal or cervical cultures, or histopathological evidence of chorioamnionitis or placental inflammation.

Control group (term deliveries): 140 mothers and their 140 corresponding term newborns. Women were selected to match the case group as closely as possible in terms of age, parity, socioeconomic and demographic status, ethnicity, place of residence, antenatal care, and year of delivery.

Inclusion criteria for the control group: delivery between 37 and 41 + 3 weeks of gestation and singleton pregnancy. All deliveries were completed naturally after uncomplicated pregnancies.

Exclusion criteria for the control group: Positive personal and/or family history of preterm birth, history of any cervical surgical procedure before or during pregnancy (e.g., conization, cerclage). As with the case group, exclusion of mothers whose newborns were stillborn or had congenital anomalies has been made. At the end, mothers and newborns with confirmed or suspected infection were excluded based on clinical presentation, elevated inflammatory markers, positive vaginal or cervical cultures, or histopathological evidence of chorioamnionitis or placental inflammation.

Preterm birth was defined according to the WHO criteria as delivery before 37 completed weeks of gestation [1,2]. Subclassification into extremely, very, and late preterm categories was not applied due to limited subgroup sizes, which would reduce statistical power. This approach was consistent with our aim to assess overall associations between *PGR* variants and spontaneous PTB.

The gestational age of each subject was determined according to the first day of the last menstrual cycle and was confirmed by ultrasound findings in the first trimester. In the case of a mismatch between the due date concerning the first day of the last menstrual cycle and the ultrasound finding, a correction to gestational age was made based on the ultrasound finding [45].

Sociodemographic, epidemiologic, and clinical data were collected in collaboration with the mothers. The available medical records on the pregnancy and delivery of the mothers were used. Data were collected on the physical condition of the mothers during pregnancy, the family and personal history of the mothers, their habits, and previous events during pregnancy.

### 4.2. Blood Sampling and Analysis

Venous blood samples were taken from pregnant women and blood from the umbilical cord of the newborns once after obtaining informed consent. Six described genetic variants of *PGR* (rs1942836, rs1042838, rs1042839, rs10895068, rs4754732, and rs653752) were analyzed. The six analyzed SNPs were selected based on previously published associations [39,40,46,47] and our earlier work in the Croatian population [11,24,25]. Although only four variants showed significant associations in our prior analysis, all six were included to evaluate their potential interaction with environmental and clinical factors. These are listed, along with their known functions, in Table 6.

The blood was taken only once: from the mothers after admission to the delivery room, in the first stage of labor; the blood of the newborns was taken from the umbilical cord vein immediately after birth. The genomic DNA was extracted from 200 µL EDTA-anticoagulated whole blood using commercially available spin columns for DNA extraction (QIAamp DNA Blood Mini Kit, Qiagen GmbH, Hilden, Germany) according to the manufacturer’s instructions [54]. The DNA samples were stored at −20 °C for further analysis.

SNP genotype analysis was performed using TaqMan-based fluorescent probes (Taq-Man SNP Genotyping Assays) [55] on the ABI PRISM 7500 Real-time PCR system (Applied Biosystems, Foster City, CA, USA). The thermocycling procedure consisted of the following: 1 hold at 95 °C for 10 min; 40 cycles of denaturation at 92 °C for 15 s; and primer annealing and extension at 60 °C for 1 min. Negative and positive control samples were run simultaneously within each analyzed real-time PCR plate. The total reaction volume per well was 25 µL with 2 µL of DNA used as a template. The allelic discrimination analysis was performed using SDS 7500 Software Version 2.3 (Applied Biosystems, Foster City, CA, USA).

### 4.3. Statistical Analysis

To detect a medium effect (0.25) in the differences in continuous variables, with a significance level of 0.05 and a power of 0.8, the minimum required sample size was 180 patients (G*Power ver. 3.1.2).

Categorical data are presented as absolute and relative frequencies. Differences in categorical variables were tested using the chi-square test and Fisher’s exact test. Normality of distribution was assessed using the Shapiro–Wilk test. Continuous data are described by medians and interquartile range limits. Differences in continuous variables between two independent groups were analyzed using the Mann–Whitney U test (Hodges–Lehmann median difference). Odds ratios (ORs) and corresponding 95% confidence intervals (CIs) were calculated for all outcomes.

An additional level of quality control for genotyping was performed using the chi-square goodness-of-fit test to compare our genotype distributions with those predicted by the Hardy–Weinberg equilibrium. Genetic association analyses were performed using logistic regression. Bonferroni correction was applied for multiple testing. All *p*-values were two-sided, with a significance level set at alpha = 0.05.

Associations between each *PGR* SNP and PTB were evaluated under three genetic inheritance models: dominant, recessive, and overdominant [29,30]. In the dominant model, carriers of at least one minor allele (heterozygous + homozygous variant genotypes) were compared with wild-type homozygotes. In the recessive model, homozygous variant genotypes were compared with carriers of at least one major allele (wild type + heterozygous genotypes). In the overdominant model, heterozygous genotypes were compared with both homozygotes combined.

Conceptually, these models represent different possible mechanisms of gene action. The dominant model assumes that one copy of the variant allele is sufficient to influence the phenotype; the recessive model assumes that two copies are required; and the overdominant model suggests that heterozygosity itself may confer a distinct, sometimes protective, effect [30].

All analyses were performed using the SNPStats web tool (http://bioinfo.iconcologia.net/SNPstats, accessed on 26 October 2025, Solé et al., 2006) [56] and MedCalc^®^ Statistical Software version 23.3.7 (MedCalc Software Ltd., Ostend, Belgium; https://www.medcalc.org; accessed on 26 October 2025), and the inheritance model showing the lowest *p*-value and most consistent odds ratio (OR) was regarded as the best-fitting model for each SNP.

## 5. Conclusions

Our findings suggest that genetic variants of the progesterone receptor gene (*PGR*), particularly rs1942836, rs4574732, and rs653752, may correlate with maternal age, fetal gender, and pregnancy complications such as vaginal bleeding to modulate the risk of spontaneous preterm birth. Their interaction with clinical and environmental factors highlights the multifactorial nature of preterm birth. These results emphasize the need for further studies in larger and more diverse populations to validate the observed associations and to explore the potential of combined genetic and clinical markers for early risk stratification and prevention strategies in obstetric care.

## Figures and Tables

**Table 1:** Characteristics of mothers and newborns born at term or prematurely.

	Median (Interquartile Range)		Difference (95% CI)	p *
	PTB (*n* = 151)	Term Birth (*n* = 141)		
Mothers’ age [years]	31 (26–36)	31 (26–34)	−1 (−2 to 1)	0.39
Weeks of gestation	34 + 6 (32 + 3–36 + 1)	39 + 3 (38 + 6–40 + 1)	4 + 5 (4 + 3 to 5 + 1)	**<0.001**
Height [cm]	165 (162–170)	166 (163–171.5)	1 (0 to 3)	0.08
Weight [kg]	73 (65–82.4)	75 (68.8–84.1)	3 (0 to 6)	**0.03**
BMI [kg/m^2^]	26.4 (24–29.6)	27.3 (24.4–30.4)	0.72 (−0.25 to 1.67)	0.14
Number of births	1 (1–2)	2 (1–2)	0 (0 to 0)	**0.03**
Number of PTB	1 (1–1)	-	-	-
Newborns weigh [g]	2440 (1830–2780)	3450 (3100–3735)	1057 (920 to 1200)	**<0.001**

* Mann–Whitney U test (Hodges–Lehmann median difference); abbreviations: BMI—body mass index; CI—confidence interval; PTB—preterm birth; bold denotes statistical significance.

**Table 2:** Frequency of clinical and environmental risk factors in the study (preterm birth < 37 weeks of gestation) and control (term birth ≥ 37 weeks) groups.

	Number (%)			p *
	PTB (*n* = 151)	Term Birth (*n* = 141)	Total (*n* = 292)	
Mothers’ age [n (%)]				
under 35 years of age	101 (67)	111 (79)	101 (67)	**0.02**
35 years of age and more	50 (33)	30 (21)	50 (33)	
Newborn’s gender [n (%)]				
Male	87 (58)	69 (49)	156 (53)	0.13
Female	64 (42)	72 (51)	136 (47)	
PTB in personal anamnesis [n (%)]				
No	127 (84)	-	127 (84)	-
Yes	24 (16)	-	24 (16)	
PTB in family anamnesis [n (%)]				
No	120 (79)	-	120 (79)	-
Yes	31 (21)	-	31 (21)	
Smoking habit [n (%)]	48 (32)	31 (22)	79 (27)	0.07
Positive cervical swab [n (%)]	31 (21)	32 (23)	63 (22)	0.65
Vaginal bleeding during pregnancy [n (%)]	41 (27)	13 (9)	54 (18)	**<0.001**

* Chi squared test; abbreviations: PTB—preterm birth; bold denotes statistical significance.

**Table 3:** Distribution and frequency of genetic inheritance models of six *PGR* SNPs in a group of prematurely born newborns, divided according to the gender of the newborn.

SNP/Inheritance Model		Genotype [*n* (%)] NEWBORNS		OR (95% CI)	*p* *
		Gender Male (*n* = 156)	Gender Female (*n* = 136)		
rs10895068 DOMINANT	G/G	138 (89)	121 (89)	1	0.99
	G/A-A/A	17 (11)	15 (11)	1.01 (0.48–2.10)	
RECESSIVE	G/G-G/A	154 (99.3)	135 (99.3)	1	0.93
	A/A	1 (0.6)	1 (0.7)	1.14 (0.07–18.41)	
OVERDOMINANT	G/G-A/A	139 (89.7)	122 (89.7)	1	0.99
	G/A	16 (10.3)	14 (10.3)	1.00 (0.47–2.13)	
rs1042838	G/G	115 (73.7)	97 (71.8)	1 1.10 (0.65–1.84)	0.72
DOMINANT	T/G-T/T	41 (26.3)	38 (28.1)		
RECESSIVE	G/G-T/G	153 (98.1)	132 (97.8)	1	0.86
	T/T	3 (1.9)	3 (2.2)	1.16 (0.23–5.84)	
OVERDOMINANT	G/G-T/T	118 (75.6)	100 (74.1)	1	0.76
	T/G	38 (24.4)	35 (25.9)	1.09 (0.64–1.85)	
rs1042839	C/C	115 (73.7)	99 (73.3)	1 1.02 (0.61–1.72)	0.94
DOMINANT	T/C-T/T	41 (26.3)	36 (26.7)		
RECESSIVE	C/C-T/C	153 (98.1)	132 (97.8)	1	0.86
	T/T	3 (1.9)	3 (2.2)	1.16 (0.23–5.84)	
OVERDOMINANT	C/C-T/T	118 (75.6)	102 (75.6)	1	0.99
	T/C	38 (24.4)	33 (24.4)	1.00 (0.59–1.72)	
rs1942836	T/T	116 (74.4)	103 (76.3)	1 0.90 (0.53–1.54)	0.70
DOMINANT	C/T-C/C	40 (25.6)	32 (23.7)		
RECESSIVE	T/T-C/T	152 (97.4)	134 (99.3)	1	0.21
	C/C	4 (2.6)	1 (0.7)	0.28 (0.03–2.57)	
OVERDOMINANT	T/T-C/C	120 (76.9)	104 (77)	1	0.98
	C/T	36 (23.1)	31 (23)	0.99 (0.57–1.72)	
rs4754732	T/T	73 (47.1)	70 (51.5)	1 0.84 (0.53–1.33)	0.46
DOMINANT	C/T-C/C	82 (52.9)	66 (48.5)		
RECESSIVE	T/T-C/T	135 (87.1)	128 (94.1)	1	**0.04**
	C/C	20 (12.9)	8 (5.9)	2.4 (1.01–5.57)	
OVERDOMINANT	T/T-C/C	93 (60)	78 (57.4)	1	0.65
	C/T	62 (40)	58 (42.6)	1.12 (0.70–1.78)	
rs653752	G/G	65 (41.7)	44 (32.6)	1 1.48 (0.91–2.39)	0.11
DOMINANT	C/G-C/C	91 (58.3)	91 (67.4)		
RECESSIVE	G/G-C/G	132 (84.6)	112 (83)	1	0.70
	C/C	24 (15.4)	23 (17)	1.13 (0.60–2.11)	
OVERDOMINANT	G/G-C/C	89 (57)	67 (49.6)	1	0.21
	C/G	67 (43)	68 (50.4)	1.35 (0.85–2.14)	

* Chi-squared test; abbreviations: SNP—single nucleotide polymorphism, *PGR*—progesterone receptor gene; OR-odds ratio; CI—confidence interval. Analyses were restricted to preterm-born newborns and were performed under dominant, recessive, and overdominant inheritance models. Odds ratios (OR) and 95% confidence intervals (CI) are presented for each model. The findings suggest potential sex-specific genetic effects of *PGR* variants among preterm deliveries. Bold denotes statistical significance.

**Table 4:** Distribution and frequency of genetic inheritance patterns of six *PGR* SNPs in a group of prematurely born newborns, divided according to maternal age.

SNP/Inheritance Model		Genotype [*n* (%)] NEWBORNS		OR (95% CI)	*p* *
		Mothers Aged Under 35 (*n* = 212)	Mothers Aged 35 and Older (*n* = 79)		
rs10895068	G/G	188 (88.7)	71 (89.9)	1 0.88 (0.38–2.06)	0.77
DOMINANT	G/A-A/A	24 (11.3)	8 (10.1)		
	G/G-G/A	210 (99.1)	79 (100)	1	0.26
RECESSIVE	A/A	2 (0.9)	0	0.00 (0.00–NA)	
	G/G-A/A	190 (89.6)	71 (89.9)	1	0.95
OVERDOMINANT	G/A	22 (10.4)	8 (10.1)	0.97 (0.41–2.29)	
	G/G	155 (73.5)	57 (71.2)	1	0.71
rs1042838	T/G-T/T	56 (26.5)	23 (28.8)	1.12 (0.63–1.98)	
DOMINANT	G/G-T/G	208 (98.6)	77 (96.2)	1	0.24
RECESSIVE	T/T	3 (1.4)	3 (3.8)	2.70 (0.53–13.67)	
	G/G-T/T	158 (74.9)	60 (75)	1	0.98
OVERDOMINANT	T/G	53 (25.1)	20 (25)	0.99 (0.55–1.80)	
	C/C	156 (73.9)	58 (72.5)	1 1.08 (0.60–1.92)	0.80
rs1042839	T/C-T/T	55 (26.1)	22 (27.5)		
DOMINANT	C/C-T/C	208 (98.6)	77 (96.2)	1	0.24
RECESSIVE	T/T	3 (1.4)	3 (3.8)	2.70 (0.53–13.67)	
	C/C-T/T	159 (75.4)	61 (76.2)	1	0.87
OVERDOMINANT	T/C	52 (24.6)	19 (23.8)	0.95 (0.52–1.74)	
	T/T	163 (77.2)	56 (70)	1	0.21
rs1942836	C/T-C/C	48 (22.8)	24 (30)	1.46 (0.82–2.59)	
DOMINANT	T/T-C/T	209 (99)	77 (96.2)	1	0.13
RECESSIVE	C/C	2 (1)	3 (3.8)	4.07 (0.67–24.83)	
	T/T-C/C	165 (78.2)	59 (73.8)	1	0.43
OVERDOMINANT	C/T	46 (21.8)	21 (26.2)	1.28 (0.70–2.32)	
	T/T	101 (47.6)	42 (53.2)	1	0.40
rs4754732	C/T-C/C	111 (52.4)	37 (46.8)	0.80 (0.48–1.35)	
DOMINANT	T/T-C/T	193 (91)	70 (88.6)	1	0.54
RECESSIVE	C/C	19 (9)	9 (11.4)	1.31 (0.56–3.02)	
	T/T-C/C	120 (56.6)	51 (64.6)	1	0.22
OVERDOMINANT	C/T	92 (43.4)	28 (35.4)	0.72 (0.42–1.22)	
	G/G	78 (37)	31 (38.8)	1	0.78
rs653752	C/G-C/C	133 (63)	49 (61.2)	0.93 (0.55–1.57)	
DOMINANT	G/G-C/G	184 (87.2)	60 (75)	1	**0.02**
RECESSIVE	C/C	27 (12.8)	20 (25)	2.27 (1.19–4.34)	
	G/G-C/C	105 (49.8)	51 (63.8)	1	**0.03**
	C/G	106 (50.2)	29 (36.2)	1.78 (1.05–3.02)	

* Chi-squared test; abbreviations: SNP—single nucleotide polymorphism, *PGR*—progesterone receptor gene; OR—odds ratio; CI—confidence interval. Analyses were performed under dominant, recessive, and overdominant inheritance models. Odds ratios (OR) and 95% confidence intervals (CI) are shown for each model. These results indicate potential age-dependent effects of *PGR* variants within the preterm-born newborns group. Bold denotes statistical significance.

**Table 5:** Distribution and frequency of genotype inheritance patterns of six *PGR* SNPs in a group of preterm newborns divided according to maternal bleeding during pregnancy.

SNP/Inheritance Model		Genotype (*n* (%)] NEWBORNS Vaginal Bleeding During Pregnancy		OR (95% CI)	*p* *
		No (*n* = 109)	Yes (*n* = 41)		
rs10895068	G/G				
DOMINANT	G/A-A/A				
	G/G-G/A				
RECESSIVE	A/A				
	G/G-A/A				
OVERDOMINANT	G/A				
	G/G	78 (71.6)	28 (68.3)	1	0.70
rs1042838	T/G-T/T	31 (28.4)	13 (31.7)	1.17 (0.54–2.54)	
DOMINANT	G/G-T/G	107 (98.2)	40 (97.6)	1	0.82
RECESSIVE	T/T	2 (1.8)	1 (2.4)	1.34 (0.12–15.16)	
	G/G-T/T	80 (73.4)	29 (70.7)	1	0.75
OVERDOMINANT	T/G	29 (26.6)	12 (29.3)	1.14 (0.52–2.53)	
	C/C	80 (73.4)	28 (68.3)	1	0.54
rs1042839	T/C-T/T	29 (26.6)	13 (31.7)	1.28 (0.59–2.80)	
DOMINANT	C/C-T/C	107 (98.2)	40 (97.6)	1	0.82
RECESSIVE	T/T	2 (1.8)	1 (2.4)	1.34 (0.12–15.16)	
	C/C-T/T	82 (75.2)	29 (70.7)	1	0.58
OVERDOMINANT	T/C	27 (24.8)	12 (29.3)	1.26 (0.56–2.80)	
	T/T	74 (67.9)	33 (80.5)	1	0.12
rs1942836	C/T-C/C	35 (32.1)	8 (19.5)	0.51 (0.21–1.22)	
DOMINANT	T/T-C/T	108 (99.1)	39 (95.1)	1	0.15
RECESSIVE	C/C	1 (0.9)	2 (4.9)	5.54 (0.49–62.80)	
	T/T-C/C	75 (68.8)	35 (85.4)	1	**0.03**
OVERDOMINANT	C/T	34 (31.2)	6 (14.6)	0.38 (0.15–0.98)	
	T/T	56 (51.4)	22 (53.7)	1	0.80
rs4754732	C/T-C/C	53 (48.6)	19 (46.3)	0.91 (0.44–1.87)	
DOMINANT	T/T-C/T	98 (89.9)	35 (85.4)	1	0.44
RECESSIVE	C/C	11 (10.1)	6 (14.6)	1.53 (0.53–4.44)	
	T/T-C/C	67 (61.5)	28 (68.3)	1	0.44
OVERDOMINANT	C/T	42 (38.5)	13 (31.7)	0.74 (0.35–1.59)	
	G/G	38 (34.9)	18 (43.9)	1	0.31
rs653752	C/G-C/C	71 (65.1)	23 (56.1)	0.68 (0.33–1.42)	
DOMINANT	G/G-C/G	92 (84.4)	35 (85.4)	1	0.88
RECESSIVE	C/C	17 (15.6)	6 (14.6)	0.93 (0.34–2.54)	
	G/G-C/C	55 (50.5)	24 (58.5)	1	0.38
OVERDOMINANT	C/G	54 (49.5)	17 (41.5)	0.72 (0.35–1.49)	

* Chi-squared test; abbreviations: SNP—single nucleotide polymorphism, *PGR*—progesterone receptor gene; OR—odds ratio; CI—confidence interval. Analyses were restricted to preterm-born newborns and performed under dominant, recessive, and overdominant inheritance models. Odds ratios (OR) and 95% confidence intervals (CI) are presented for each model. These findings may reflect gene–environment interactions between *PGR* variants and clinical complications/vaginal bleeding in preterm pregnancies. Bold denotes statistical significance.

**Table 6:** Studied progesterone receptor single-nucleotide polymorphisms (SNPs).

SNP	Location	Gene Region	Base Change	Citation	Related Phenotype
rs1942836	chr11:101178616 (GRCh38.p14)	Potential promoter region	T/C>T/G	Bahia et al. [46] Mann et al. [47] Hackbart et al. [48] Ehn et al. [39]	Habitual abortions Preterm birth
rs1042828	chr11:101062681 (GRCh38.p14)	Missense mutation Exon 4	C/A>C/G	Ghali et al. [49] Pearce et al. [50] Khan et al. [51] Ehn et al. [39] Luo et al. [44]	Ovary cancer Breast cancer Habitual abortions Preterm birth
rs1042839	hr11:101051471 (GRCh38.p14)	Missense mutation Exon 5	G>A	Ghali et al. [49] Pearce et al. [50] Khan et al. [51] Ehn et al. [39] Luo et al. [44]	Ovary cancer Breast cancer Habitual abortions Preterm birth
rs10895068	chr11:101,129,483 (GRCh38.p14)	5′ UTR region	GC>T	Bahia et al. [46] Ghali et al. [49] Mulac-Jericević et al. [52] Ehn et al. [39]	Endometrial cancer Ovary cancer Preterm birth
rs4754732	chr11:101.137.771 (GRCh38.p14)	*PGR*-AS1: intron region	T/A>T/C	Ehn et al. [39] Manuck et al. [40]	Preterm birth
rs653752	chr11:101.077.379 (GRCh38.p14)	Intron region	C>G	Ehn et al. [39] Bustos et al. [53] Mann et al. [47] Manuck et al. [40]	Preterm birth

## Data Availability

The original contributions presented in this study are included in the article/supplementary material. Further inquiries can be directed to the corresponding author.

## Supplementary Materials

The following supporting information can be downloaded at: https://www.mdpi.com/article/10.3390/ijms262110659/s1.

## Abbreviations

The following abbreviations are used in this manuscript:

ANOVA	Analysis of Variance
BMI	Body Mass Index
CI	Confidence Interval
DNA	Deoxyribonucleic Acid
FPW	Functional Progesterone Withdrawal
*GJA1*	Gap Junction Protein Alpha 1 gene
HWE	Hardy–Hardy–Weinberg Equilibrium
IL-1	Interleukin-1
NF-κB2	Nuclear Factor κB Subunit 2
OR	Odds Ratio
*OXTR*	Oxytocin Receptor gene
P4	Progesterone
PCR	Polymerase Chain Reaction
*PGR*	Progesterone Receptor (gene)
*PGR*-AS1	Progesterone Receptor Antisense RNA 1(gene)
PPROM	Preterm Premature Rupture of Membranes
PR	Progesterone Receptor (protein)
PR-A	Progesterone Receptor Isoform A
PR-B	Progesterone Receptor Isoform B
PTB	Preterm Birth
*PTGS2*	Prostaglandin-Endoperoxide Synthase 2 (*COX-2*) gene
RNA	Ribonucleic Acid
SD	Standard Deviation
SNP	Single Nucleotide Polymorphism
TLR-4	Toll-Like Receptor 4
TNFα	Tumor Necrosis Factor alpha
WHO	World Health Organization
