# Supplementary material for: Genetic and Environmental Determinants of Spontaneous Preterm Birth: Focus on Progesterone Receptor Gene Variants
Source: Int J Mol Sci. 2025 Nov 1;26(21):10659. doi: 10.3390/ijms262110659 (PMC12609070; doi:10.3390/ijms262110659)
Supplement: Supplementary file 1 [file ijms-26-10659-s001.zip › ijms-3928052-supplementary.pdf]

Table S1. Genotype distribution and allele frequencies of six selected *PGR* SNPs in mothers with premature birth and the respective controls, when divided into two subgroups considering newborn gender.

Single Nucleotide Polymorphisms		**Mothers** Genotype [n (%)] Newborns Gender		Odds ratio (95% Confidence interval)	*P**
		Male	Female		
rs10895068	G/G	131 (84)	120 (88,2)	1	0,55
	G/A	24 (15,4)	15 (11)	0,68 (0,34-1,36)	
	A/A	1 (0,6)	1 (0,7)	1,09 (0,07-17,65)	
Allele	**G**	286 (92)	255 (94)	0,73 (0,39 – 1,38)	0,34
	**A**	26 (8)	17 (6)		
rs1042838	G/G	117 (75)	103 (75,7)	1	0,80
	T/G	35 (22,4)	31 (22,8)	1,01 (0,58-1,75)	
	T/T	4 (2,6)	2 (1,5)	0,57 (0,10-3,17)	
Allele	**G**	269 (86)	237 (87)	0,92 (0,57 – 1,49)	0,75
	**T**	43 (14)	35 (13)		
rs1042839	C/C	117 (75)	105 (77,2)	1	0,77
	T/C	35 (22,4)	29 (21,3)	0,92 (0,53-1,61)	
	T/T	4 (2,6)	2 (1,5)	0,56 (0,10-3,10)	
Allele	**C**	269 (86)	239 (88)	0,86 (0,53 – 1,4)	0,55
	**T**	43 (14)	33 (12)		
rs1942836	T/T	104 (66,7)	97 (71,3)	1	0,51
	C/T	49 (31,4)	35 (25,7)	0,77 (0,46-1,28)	
	C/C	3 (1,9)	4 (2,9)	1,43 (0,31-6,55)	
Allele	**C**	55 (18)	43 (16)	0,87 (0,57 – 1,36)	0,56
	**T**	257 (82)	229 (84)		
rs4754732	T/T	79 (50.6)	71 (52.2)	1.00	0.86
	C/T	64 (41)	52 (38.2)	0.90 (0.56-1.47)	
	C/C	13 (8.3)	13 (9.6)	1.11 (0.48-2.56)	
Allele	**T**	222 (71)	194 (71)	0,99 (0,69 – 1,4)	0,96
	**C**	90 (29)	78 (29)		
rs653752	G/G	61 (39,1)	53 (39)	1	0,65
	C/G	69 (44,2)	55 (40,4)	0,92 (0,55-1,53)	
	C/C	26 (16,7)	28 (20,6)	1,24 (0,65-2,37)	
Allele	**C**	191 (61)	161 (59)	0,82 (0,66 – 1,3)	0,62
	**G**	121 (39)	111 (41)		

* chi-squared test; SNP-.single nucleotide polymorphism, *PGR*-progesterone receptor gene

Table S2. Models of inheritance and their frequencies of six selected PGR SNPs in mothers with premature birth and the respective controls, when divided into two subgroups considering newborn gender.

		Genotype [n (%)] **Mothers**		Odds ratio (95% Confidence interval)	*P**
		**Newborns Gender Male**	**Newbons Gender Female**		
rs10895068 DOMINANT	G/G	131 (84)	120 (88,2)	1	0,29
	G/A-A/A	25 (16)	16 (11,8)	0,70 (0,36-1,37)	
RECESSIVE	G/G-G/A	155 (99,4)	135 (99,3)	1	0,92
	A/A	1 (0,6)	1 (0,7)	1,15 (0,07-18,53)	
OVERDOMINANT	G/G-A/A	132 (84,6)	121 (89)	1	0,27
	G/A	24 (15,4)	15 (11)	0,68 (0,34-1,36)	
rs1042838 DOMINANT	G/G	117 (75)	103 (75,7)	1	0,88
	T/G-T/T	39 (25)	33 (24,3)	0,96 (0,56-1,64)	
RECESSIVE	G/G-T/G	152 (97,4)	134 (98,5)	1	0,51
	T/T	4 (2,6)	2 (1,5)	0,57 (0,10-3,15)	
OVERDOMINANT	G/G-T/T	121 (77,6)	105 (77,2)	1	0,94
	T/G	35 (22,4)	31 (22,8)	1,02 (0,59-1,77)	
rs1042839 DOMINANT	C/C	117 (75)	105 (77,2)	1	0,66
	T/C-T/T	39 (25)	31 (22,8)	0,89 (0,52-1,52)	
RECESSIVE	C/C-T/C	152 (97,4)	134 (98,5)	1	0,51
	T/T	4 (2,6)	2 (1,5)	0,57 (0,10-3,15)	
OVERDOMINANT	C/C-T/T	121 (77,6)	107 (78,7)	1	0,82
	T/C	35 (22,4)	29 (21,3)	0,94 (0,54-1,63)	
rs1942836 DOMINANT	T/T	104 (66,7)	97 (71,3)	1	0,39
	C/T-C/C	52 (33,3)	39 (28,7)	0,80 (0,49-1,32)	
RECESSIVE	T/T-C/T	153 (98,1)	132 (97,1)	1	0,57
	C/C	3 (1,9)	4 (2,9)	1,55 (0,34-7,03)	
OVERDOMINANT	T/T-C/C	107 (68,6)	101 (74,3)	1	0,28
	C/T	49 (31,4)	35 (25,7)	0,76 (0,45-1,26)	
rs4754732 DOMINANT	T/T	79 (50.6%)	71 (52.2%)	1.00	0.79
	C/T-C/C	77 (49.4%)	65 (47.8%)	0.94 (0.59-1.49)	
RECESSIVE	T/T-C/T	143 (91.7%)	123 (90.4%)	1.00	0.71
	C/C	13 (8.3%)	13 (9.6%)	1.16 (0.52-2.60)	
OVERDOMINANT	T/T-C/C	92 (59%)	84 (61.8%)	1.00	0.63
	C/T	64 (41%)	52 (38.2%)	0.89 (0.56-1.42)	
rs653752 DOMINANT	G/G	61 (39,1)	53 (39)	1	0,98
	C/G-C/C	95 (60,9)	83 (61)	1,01 (0,63-1,61)	
RECESSIVE	G/G-C/G	130 (83,3)	108 (79,4)	1	0,39
	C/C	26 (16,7)	28 (20,6)	1,30 (0,72-2,34)	
OVERDOMINANT	G/G-C/C	87 (55,8)	81 (59,6)	1	0,51
	C/G	69 (44,2)	55 (40,4)	0,86 (0,54-1,36)	

* chi-squared test; SNP-.single nucleotide polymorphism, *PGR*-progesterone receptor gene

Table S 3. Genotype distribution and allele frequencies of six selected *PGR* SNPs in mothers with premature birth and the respective controls, when divided into two subgroups considering mothers' age.

Single Nucleotide Polymorphisms		**Mothers**			
		Genotype [n (%)] Mothers age		Odds ratio (95% Confidence interval)	*P**
		Under 35 years of age	Over 35 years of age		
rs10895068	G/G	179 (84,4)	72 (90)	1	0,29
	G/A	31 (14,6)	8 (10)	0,64 (0,28-1,46)	
	A/A	2 (0,9)	0	-	
Allele	**G**	389 (92)	152 (95)	0,59 (0,27 – 1,29)	0,18
	**A**	35 (8)	8 (5)		
rs1042838	G/G	162 (76,4)	58 (72,5)	1	0,46
	T/G	47 (22,2)	19 (23,8)	1,13 (0,61-2,08)	
	T/T	3 (1,4)	3 (3,8)	2,79 (0,55-14,23)	
Allele	**G**	371 (88)	135 (84)	1,29 (0,77 – 2,17)	0,32
	**T**	53 (12)	25 (16)		
rs1042839	C/C	163 (76,9)	59 (73,8)	1	0,48
	T/C	46 (21,7)	18 (22,5)	1,08 (0,58-2,01)	
	T/T	3 (1,4)	3 (3,8)	2,76 (0,54-14,07)	
	**C**	372 (88)	136 (85)	1,26 (0,75 – 2,13)	0,38
Allele	**T**	52 (12)	24 (15)		
	T/T	147 (69,3)	54 (67,5)	1	0,96
rs1942836	C/T	60 (28,3)	24 (30)	1,09 (0,62-1,92)	
	C/C	5 (2,4)	2 (2,5)	1,09 (0,21-5,78)	
	**T**	354 (83)	132 (82)	1,07 (0,66 – 1,74)	0,78
Allele	**C**	70 (17)	28 (18)		
	T/T	109 (51,4)	41 (51,2)	1	0,56
rs4754732	C/T	82 (38,7)	34 (42,5)	1,10 (0,64-1,89)	
	C/C	21 (9,9)	5 (6,2)	0,63 (0,22-1,79)	
	**T**	300 (71)	116 (72)	0,92 (0,61 – 1,38)	0,68
Allele	**C**	124 (29)	44 (28)		
	G/G	85 (40,1)	29 (36,2)	1	0,72
rs653752	C/G	87 (41)	37 (46,2)	1,25 (0,70-2,21)	
	C/C	40 (18,9)	14 (17,5)	1,03 (0,49-2,15)	
	**G**	257 (61)	95 (59)	1,05 (0,73 – 1,53)	0,79
Allele	**C**	167 (39)	65 (41)		

* chi-squared test; SNP-.single nucleotide polymorphism, *PGR*-progesterone receptor gene

Table S 4. Models of inheritance and their frequencies of six selected *PGR* SNPs in mothers with premature birth and the respective controls, when divided into two subgroups based on maternal age.

		Genotype [n (%)] **Mothers**		Odds ratio (95% Confidence interval)	*P**
		Under 35 years of age	Over 35 years of age		
rs10895068	**G/G**	179 (84,4)	72 (90)	1	0,21
DOMINANT	**G/A-A/A**	33 (15,6)	8 (10)	0,60 (0,27-1,37)	
	**G/G-G/A**	210 (99,1)	80 (100)	1	0,26
RECESSIVE	**A/A**	2 (0,9)	0	-	
	**G/G-A/A**	181 (85,4)	72 (90)	1	0,29
OVERDOMINANT	**G/A**	31 (14,6)	8 (10)	0,65 (0,28-1,48)	
	**G/G**	162 (76,4)	58 (72,5)	1	0,49
rs1042838	**T/G-T/T**	50 (23,6)	22 (27,5)	1,23 (0,69-2,20)	
DOMINANT	**G/G-T/G**	209 (98,6)	77 (96,2)	1	0,24
RECESSIVE	**T/T**	3 (1,4)	3 (3,8)	2,71 (0,54-13,74)	
	**G/G-T/T**	165 (77,8)	61 (76,2)	1	0,77
OVERDOMINANT	**T/G**	47 (22,2)	19 (23,8)	1,09 (0,60-2,01)	
	**C/C**	163 (76,9)	59 (73,8)	1	0,58
rs1042839	**T/C-T/T**	49 (23,1)	21 (26,2)	1,18 (0,66-2,14)	
DOMINANT	**C/C-T/C**	209 (98,6)	77 (96,2)	1	0,24
RECESSIVE	**T/T**	3 (1,4)	3 (3,8)	2,71 (0,54-13,74)	
	**C/C-T/T**	166 (78,3)	62 (77,5)	1	0,88
OVERDOMINANT	**T/C**	46 (21,7)	18 (22,5)	1,05 (0,56-1,94)	
	**T/T**	147 (69,3)	54 (67,5)	1	0,76
rs1942836	**C/T-C/C**	65 (30,7)	26 (32,5)	1,09 (0,63-1,89)	
DOMINANT	**T/T-C/T**	207 (97,6)	78 (97,5)	1	0,94
RECESSIVE	**C/C**	5 (2,4)	2 (2,5)	1,06 (0,20-5,59)	
	**T/T-C/C**	152 (71,7)	56 (70)	1	0,78
OVERDOMINANT	**C/T**	60 (28,3)	24 (30)	1,09 (0,62-1,91)	
	**T/T**	109 (51,4)	41 (51,2)	1	0,98
rs4754732	**C/T-C/C**	103 (48,6)	39 (48,8)	1,01 (0,60-1,68)	
DOMINANT	**T/T-C/T**	191 (90,1)	75 (93,8)	1	0,31
RECESSIVE	**C/C**	21 (9,9)	5 (6,2)	0,61 (0,22-1,67)	
	**T/T-C/C**	130 (61,3)	46 (57,5)	1	0,55
OVERDOMINANT	**C/T**	82 (38,7)	34 (42,5)	1,17 (0,69-1,98)	
	**G/G**	85 (40,1)	29 (36,2)	1	0,55
rs653752	**C/G-C/C**	127 (59,9)	51 (63,8)	1,18 (0,69-2,00)	
DOMINANT	**G/G-C/G**	172 (81,1)	66 (82,5)	1	0,79
RECESSIVE	**C/C**	40 (18,9)	14 (17,5)	0,91 (0,47-1,79)	
OVERDOMINANT	**G/G-C/C**	125 (59)	43 (53,8)	1	0,42
	**C/G**	87 (41)	37 (46,2)	1,24 (0,74-2,07)	

* chi-squared test; SNP-.single nucleotide polymorphism, *PGR*-progesterone receptor gene

Table S 5. Genotype distribution and allele frequencies of six selected *PGR* SNPs in mothers with premature birth and the respective controls, when divided into two subgroups considering preterm birth in mothers' personal anamnesis.

Single Nucleotide Polymorphisms		**Mothers** Genotype [n (%)] Preterm birth in personal anamnesis		Odds ratio (95% Confidence interval)	*P**
		No	Yes		
rs10895068	**AA**	0	0		
	**GA**	20 (16)	2 (8)	0,49 (0,11 – 2,23)	0,53
	**GG**	107 (84)	22 (92)	1,0	
Allele	**A**	20 (8)	2 (4)	1,97 (0,44 – 8,70)	0,37
	**G**	234 (92)	46 (96)		
rs1042838	**GG**	93 (73)	16 (67)	1,0	0,18
	**TG**	32 (25)	6 (25)	1,09 (0,39 – 3,02)	
	**TT**	2 (2)	2 (8)	5,81 (0,76 – 44,28)	
Allele	**G**	218 (86)	38 (79)	0,63 (0,29 – 1,37)	0,24
	**T**	36 (14)	10 (21)		
rs1042839	**CC**	95 (75)	16 (67)	1,0	0,15
	**TC**	30 (24)	6 (25)	1,30 (0,46 – 3,63)	
	**TT**	2 (2)	2 (8)	6,06 (0,80 – 46,16)	
Allele	**C**	220 (87)	38 (79)	0,53 (0,27 – 1,22)	0,13
	**T**	34 (13)	10 (21)		
rs1942836	**CC**	5 (4)	0	-	0,84
	**CT**	35 (28)	6 (25)	0,83 (0,30 – 2,26)	
	**TT**	87 (69)	18 (75)	1,0	
Allele	**C**	209 (82)	42 (88)	1,51 (0,60 – 3,76)	0,38
	**T**	45 (18)	6 (12)		
rs4754732	**CC**	14 (11)	2 (8)	0,69 (0,14 – 3,42)	0,95
	**CT**	50 (39)	9 (38)	0,87 (0,35 – 2,21)	
	**TT**	63 (50)	13 (54)	1,0	
Allele	**T**	176 (69)	35 (73)	1,19 (0,59 – 2,37)	0,62
	**C**	78 (31)	13 (27)		
rs653752	**CC**	19 (15)	4 (17)	1,65 (0,44 – 6,28)	0,43
	**CG**	53 (42)	13 (54)	1,93 (0,71 – 5,20)	
	**GG**	55 (43)	7 (29)	1,0	
Allele	**C**	163 (64)	27 (56)	0,72 (0,38 – 1,34)	0,30
	**G**	91 (36)	21 (44)		

* chi-squared test; SNP-.single nucleotide polymorphism, *PGR*-progesterone receptor gene

Table S 6. Models of inheritance and their frequencies of six selected *PGR* SNPs in mothers with premature birth and the respective controls, when divided into two subgroups based on preterm birth in mothers' personal anamnesis.

		Genotype [n (%)] **Mothers** Preterm birth in the personal anamnesis of mothers		Odds ratio (95% Confidence interval)	*P**
		**No**	**Yes**		
rs10895068	**G/G**				
DOMINANT	**G/A-A/A**				
RECESSIVE	**G/G-G/A**				
	**A/A**				
OVERDOMINANT	**G/G-A/A**				
	**G/A**				
rs1042838	**G/G**	93 (73,2)	16 (66,7)	1	0,52
DOMINANT	**T/G-T/T**	34 (26,8)	8 (33,3)	1,37 (0,54-3,48)	
RECESSIVE	**G/G-T/G**	125 (98,4)	22 (91,7)	1	0,11
	**T/T**	2 (1,6)	2 (8,3)	5,68 (0,76-42,48)	
OVERDOMINANT	**G/G-T/T**	95 (74,8)	18 (75)	1	0,98
	**T/G**	32 (25,2)	6 (25)	0,99 (0,36-2,71)	
rs1042839	**C/C**	95 (74,8)	16 (66,7)	1	0,42
DOMINANT	**T/C-T/T**	32 (25,2)	8 (33,3)	1,48 (0,58-3,79)	
RECESSIVE	**C/C-T/C**	125 (98,4)	22 (91,7)	1	0,11
	**T/T**	2 (1,6)	2 (8,3)	5,68 (0,76-42,48)	
OVERDOMINANT	**C/C-T/T**	97 (76,4)	18 (75)	1	0,88
	**T/C**	30 (23,6)	6 (25)	1,08 (0,39-2,96)	
rs1942836	**T/T**	87 (68,5)	18 (75)	1	0,52
DOMINANT	**C/T-C/C**	40 (31,5)	6 (25)	0,73 (0,27-1,96)	
RECESSIVE	**T/T-C/T**	122 (96,1)	24 (100)	1	0,18
	**C/C**	5 (3,9)	0	0,00 (0,00-NA)	
OVERDOMINANT	**T/T-C/C**	92 (72,4)	18 (75)	1	0,79
	**C/T**	35 (27,6)	6 (25)	0,88 (0,32-2,39)	
rs4754732	**T/T**	63 (49,6)	13 (54,2)	1	0,68
DOMINANT	**C/T-C/C**	64 (50,4)	11 (45,8)	0,83 (0,35-2,00)	
RECESSIVE	**T/T-C/T**	113 (89)	22 (91,7)	1	0,69
	**C/C**	14 (11)	2 (8,3)	0,73 (0,16-3,46)	
OVERDOMINANT	**T/T-C/C**	77 (60,6)	15 (62,5)	1	0,86
	**C/T**	50 (39,4)	9 (37,5)	0,92 (0,38-2,27)	
rs653752	**G/G**	55 (43,3)	7 (29,2)	1	0,19
DOMINANT	**C/G-C/C**	72 (56,7)	17 (70,8)	1,86 (0,72-4,79)	
RECESSIVE	**G/G-C/G**	108 (85)	20 (83,3)	1	0,83
	**C/C**	19 (15)	4 (16,7)	1,14 (0,35-3,70)	
OVERDOMINANT	**G/G-C/C**	74 (58,3)	11 (45,8)	1	0,26

	**C/G**	53 (41,7)	13 (54,2)	1,65 (0,69-3,97)	

* chi-squared test; SNP-.single nucleotide polymorphism, *PGR*-progesterone receptor gene

Table S 7. Genotype distribution and allele frequencies of six selected *PGR* SNPs in mothers with premature birth, when divided into two subgroups considering preterm birth in mothers' family anamnesis.

		**Mothers**			
		Genotype [n (%)] Preterm birth in family anamnesis		Odds ratio (95% Confidence interval)	*P**
		No	yes		
rs10895068	**AA**	0	0	-	0,78
	**GA**	17 (14)	5 (16)	1,17 (0,39 – 3,45)	
	**GG**	103 (86)	26 (84)	1,0	
Allele	**G**	223 (93)	57 (92)	0,87 (0,31 – 2,46)	0,79
	**A**	17 (7)	5 (8)		
rs1042838	**GG**	88 (73)	21 (68)	1,0	0,71
	**TG**	29 (24)	9 (29)	1,30 (0,54 – 3,16)	
	**TT**	3 (3)	1 (3)	1,40 (0,14 – 14,11)	
Allele	**G**	205 (85)	51 (82)	0,79 (0,37 – 1,67)	0,54
	**T**	35 (15)	11 (18)		
rs1042839	**CC**	90 (75)	21 (68)	1,0	0,47[†]
	**TC**	27 (22)	9 (29)	1,58 (0,64 – 3,87)	
	**TT**	3 (3)	1 (3)	1,46 (0,14 – 14,75)	
Allele	**C**	207 (87)	51 (82)	0,70 (0,32 – 1,56)	0,32
	**T**	33 (13)	11 (18)		
rs1942836	**CC**	4 (3)	1 (3)	0,94 (0,10 – 8,87)	>0,99
	**CT**	33 (28)	8 (26)	0,91 (0,37 – 2,26)	
	**TT**	83 (69)	22 (71)	1,0	
Allele	**T**	199 (83)	52 (84)	1,07 (0,50 – 2,28)	0,86
	**C**	41 (17)	10 (16)		
rs4754732	**CC**	13 (11)	3 (10)	0,80 (0,20 – 3,14)	0,92
	**CT**	48 (40)	11 (35)	0,80 (0,34 – 1,86)	
	**TT**	59 (49)	17 (55)	1,0	
Allele	**T**	166 (69)	45 (73)	1,18 (0,63 – 2,19)	0,60
	**C**	74 (31)	17 (27)		
rs653752	**CC**	18 (15)	5 (16)	0,95 (0,30 – 3,03)	0,83
	**CG**	54 (45)	12 (39)	0,76 (0,32 – 1,81)	
	**GG**	48 (40)	14 (45)	1,0	
Allele	**G**	150 (62)	40 (65)	1,09 (0,61 – 1,95)	0,77
	**C**	90 (38)	22 (35)		

* chi-squared test; SNP-.single nucleotide polymorphism, *PGR*-progesterone receptor gene

Table S 8. Models of inheritance and their frequencies of six selected *PGR* SNPs in mothers with premature birth and the respective controls, when divided into two subgroups based on preterm birth in mothers' family anamnesis.

		Genotype [n (%)] **Mothers** Preterm birth in family anamnesis		Odds ratio (95% Confidence interval)	*P**
		**No**	**Yes**		
rs10895068	**G/G**				
DOMINANT	**G/A-A/A**				
RECESSIVE	**G/G-G/A**				
	**A/A**				
OVERDOM	**G/G-A/A**				
	**G/A**				
rs1042838	**G/G**	88 (73,3)	21 (67,7)	1	0,54
DOMINANT	**T/G-T/T**	32 (26,7)	10 (32,3)	1,31 (0,56-3,08)	
RECESSIVE	**G/G-T/G**	117 (97,5)	30 (96,8)	1	0,83
	**T/T**	3 (2,5)	1 (3,2)	1,30 (0,13-12,95)	
OVERDOM	**G/G-T/T**	91 (75,8)	22 (71)	1	0,58
	**T/G**	29 (24,2)	9 (29)	1,28 (0,53-3,10)	
rs1042839	**C/C**	90 (75)	21 (67,7)	1	0,42
DOMINANT	**T/C-T/T**	30 (25)	10 (32,3)	1,43 (0,61-3,37)	
RECESSIVE	**C/C-T/C**	117 (97,5)	30 (96,8)	1	0,83
	**T/T**	3 (2,5)	1 (3,2)	1,30 (0,13-12,95)	
OVERDOM	**C/C-T/T**	93 (77,5)	22 (71)	1	0,45
	**T/C**	27 (22,5)	9 (29)	1,41 (0,58-3,42)	
rs1942836	**T/T**	83 (69,2)	22 (71)	1	0,85
DOMINANT	**C/T-C/C**	37 (30,8)	9 (29)	0,92 (0,39-2,18)	
RECESSIVE	**T/T-C/T**	116 (96,7)	30 (96,8)	1	0,98
	**C/C**	4 (3,3)	1 (3,2)	0,97 (0,10-8,97)	
OVERDOMINANT	**T/T-C/C**	87 (72,5)	23 (74,2)	1	0,85
	**C/T**	33 (27,5)	8 (25,8)	0,92 (0,37-2,25)	
rs4754732	**T/T**	59 (49,2)	17 (54,8)	1	0,57
DOMINANT	**C/T-C/C**	61 (50,8)	14 (45,2)	0,80 (0,36-1,76)	
RECESSIVE	**T/T-C/T**	107 (89,2)	28 (90,3)	1	0,85
	**C/C**	13 (10,8)	3 (9,7)	0,88 (0,23-3,31)	
OVERDOMINANT	**T/T-C/C**	72 (60)	20 (64,5)	1	0,64
	**C/T**	48 (40)	11 (35,5)	0,82 (0,36-1,88)	
rs653752	**G/G**	48 (40)	14 (45,2)	1	0,60
DOMINANT	**C/G-C/C**	72 (60)	17 (54,8)	0,81 (0,37-1,79)	
RECESSIVE	**G/G-C/G**	102 (85)	26 (83,9)	1	0,88
	**C/C**	18 (15)	5 (16,1)	1,09 (0,37-3,21)	
OVERDOMINANT	**G/G-C/C**	66 (55)	19 (61,3)	1	0,53
	**C/G**	54 (45)	12 (38,7)	0,77 (0,34-1,73)	

* chi-squared test; SNP-.single nucleotide polymorphism, *PGR*-progesterone receptor gene

Table S 9. Genotype distribution and allele frequencies of six selected *PGR* SNPs in mothers with premature birth, when divided into two subgroups considering mothers' smoking habits.

		**Mothers**			
		Genotype [n (%)] Smoking habits		Odds ratio (95% Confidence interval)	*P*
		No	Yes		
rs10895068	**AA**	0	0	-	0,63
	**GA**	14 (14)	8 (17)	1,27 (0,49 – 3,27)	
	**GG**	89 (86)	40 (83)	1,0	
Allele	**G**	192 (93)	88 (92)	0,80 (0,32 – 1,98)	0,63*
	**A**	14 (7)	8 (8)		
rs1042838	**GG**	75 (73)	34 (71)	1,0	0,94
	**TG**	25 (24)	13 (27)	1,15 (0,52 – 2,51)	
	**TT**	3 (3)	1 (2)	0,74 (0,07 – 7,33)	
Allele	**G**	175 (85)	81 (84)	0,96 (0,49 – 1,87)	0,90*
	**T**	31 (15)	15 (16)		
rs1042839	**CC**	76 (74)	36 (75)	1,0	>0,99
	**TC**	24 (23)	11 (23)	1,02 (0,45 – 2,32)	
	**TT**	3 (3)	1 (2)	0,71 (0,07 – 7,09)	
Allele	**C**	176 (85)	82 (85)	0,96 (0,47 – 1,93)	0,90*
	**T**	30 (15)	14 (15)		
rs1942836	**CC**	5 (5)	0	-	0,39
	**CT**	28 (27)	13 (27)	0,93 (0,43 – 2,01)	
	**TT**	70 (68)	35 (73)	1,0	
Allele	**T**	168 (82)	83 (86)	1,44 (0,73 – 2,86)	0,29*
	**C**	38 (18)	13 (14)		
rs4754732	**CC**	9 (9)	7 (15)	2,18 (0,72 – 6,62)	0,29
	**CT**	38 (37)	21 (44)	1,55 (0,74 – 3,24)	
	**TT**	56 (54)	20 (42)	1,0	
Allele	**T**	150 (73)	61 (64)	0,65 (0,39 – 1,09)	0,10*
	**C**	56 (27)	35 (36)		
rs653752	**CC**	17 (17)	6 (13)	0,64 (0,22 – 1,86)	0,69
	**CG**	46 (45)	20 (42)	0,79 (0,38 – 1,66)	
	**GG**	40 (39)	22 (46)	1,0	
Allele	**G**	126 (61)	64 (67)	1,27 (0,76 – 2,11)	0,36*
	**C**	80 (39)	32 (33)		

* chi-squared test; SNP-.single nucleotide polymorphism, *PGR*-progesterone receptor gene

Table S 10. Models of inheritance and their frequencies of six selected *PGR* SNPs in mothers with premature birth and the respective controls, when divided into two subgroups based on mothers' smoking habits.

		Genotype [n (%)] **Mothers** Smoking Habit		Odds ratio (95% Confidence interval)	*P**
		**No**	**Yes**		
rs10895068 DOMINANT	**G/G**				
	**G/A-A/A**				
RECESSIVE	**G/G-G/A**				
	**A/A**				
OVERDOMINANT	**G/G-A/A**				
	**G/A**				
rs1042838 DOMINANT	**G/G**	75 (72,8)	34 (70,8)	1	0,80
	**T/G-T/T**	28 (27,2)	14 (29,2)	1,10 (0,52-2,36)	
RECESSIVE	**G/G-T/G**	100 (97,1)	47 (97,9)	1	0,76
	**T/T**	3 (2,9)	1 (2,1)	0,71 (0,07-7,00)	
OVERDOMINANT	**G/G-T/T**	78 (75,7)	35 (72,9)	1	0,71
	**T/G**	25 (24,3)	13 (27,1)	1,16 (0,53-2,53)	
rs1042839 DOMINANT	**C/C**	76 (73,8)	35 (72,9)	1	0,91
	**T/C-T/T**	27 (26,2)	13 (27,1)	1,05 (0,48-2,27)	
RECESSIVE	**C/C-T/C**	100 (97,1)	47 (97,9)	1	0,76
	**T/T**	3 (2,9)	1 (2,1)	0,71 (0,07-7,00)	
OVERDOMINANT	**C/C-T/T**	79 (76,7)	36 (75)	1	0,82
	**T/C**	24 (23,3)	12 (25)	1,10 (0,49-2,44)	
rs1942836 DOMINANT	**T/T**	70 (68)	35 (72,9)	1	0,54
	**C/T-C/C**	33 (32)	13 (27,1)	0,79 (0,37-1,68)	
RECESSIVE	**T/T-C/T**	98 (95,2)	48 (100)	1	0,048
	**C/C**	5 (4,8)	0	-	
OVERDOMINANT	**T/T-C/C**	75 (72,8)	35 (72,9)	1	0,99
	**C/T**	28 (27,2)	13 (27,1)	0,99 (0,46-2,15)	
rs4754732 DOMINANT	**T/T**	56 (54,4)	20 (41,7)	1	0,15
	**C/T-C/C**	47 (45,6)	28 (58,3)	1,67 (0,83-3,33)	
RECESSIVE	**T/T-C/T**	94 (91,3)	41 (85,4)	1	0,29
	**C/C**	9 (8,7)	7 (14,6)	1,78 (0,62-5,11)	
OVERDOMINANT	**T/T-C/C**	65 (63,1)	27 (56,2)	1	0,42
	**C/T**	38 (36,9)	21 (43,8)	1,33 (0,66-2,67)	
rs653752 DOMINANT	**G/G**	40 (38,8)	22 (45,8)	1	0,42
	**C/G-C/C**	63 (61,2)	26 (54,2)	0,75 (0,38-1,50)	
RECESSIVE	**G/G-C/G**	86 (83,5)	42 (87,5)	1	0,52
	**C/C**	17 (16,5)	6 (12,5)	0,72 (0,27-1,97)	
OVERDOMINANT	**G/G-C/C**	57 (55,3)	28 (58,3)	1	0,73
	**C/G**	46 (44,7)	20 (41,7)	0,89 (0,44-1,77)	

* chi-squared test; SNP-.single nucleotide polymorphism, *PGR*-progesterone receptor gene

Table S 11. Genotype distribution and allele frequencies of six selected *PGR* SNPs in mothers with premature birth, when divided into two subgroups considering mothers' BMI

		**Mothers**			
		Genotype [n (%)] BMI		Odds ratio (95% Confidence interval)	*P**
		Normal	Overweight		
rs10895068	**AA**	0	0	-	0,48
	**GA**	10 (18)	12 (13)	0,69 (0,28 – 1,71)	
	**GG**	47 (82)	82 (87)	1,0	
Allele	**G**	104 (91)	176 (94)	1,41 (0,59 – 3,38)	0,44
	**A**	10 (9)	12 (6)		
rs1042838	**GG**	44 (77)	65 (69)	1,0	0,23†
	**TG**	13 (23)	25 (27)	1,30 (0,60 – 2,82)	
	**TT**	0	4 (4)	-	
Allele	**G**	101 (89)	155 (82)	0,60 (0,30 – 1,20)	0,15
	**T**	13 (11)	33 (18)		
rs1042839	**CC**	45 (79)	66 (70)	1,0	0,11
	**TC**	12 (21)	24 (26)	1,36 (0,62 – 3,0)	
	**TT**	0	4 (4)	-	
Allele	**C**	102 (89)	158 (84)	0,62 (0,30 – 1,27)	0,19
	**T**	12 (11)	30 (16)		
rs1942836	**CC**	2 (4)	3 (3)	0,85 (0,14 – 5,32)	0,84
	**CT**	17 (30)	24 (26)	0,80 (0,38 – 1,67)	
	**TT**	38 (67)	67 (71)	1,0	
Allele	**T**	93 (82)	158 (84)	1,19 (0,64 – 2,19)	0,58
	**C**	21 (18)	30 (16)		
rs4754732	**CC**	7 (12)	9 (10)	0,79 (0,27 – 2,36)	0,83
	**CT**	21 (37)	38 (40)	1,12 (0,55 – 2,26)	
	**TT**	29 (51)	47 (50)	1,0	
Allele	**T**	79 (69)	132 (70)	1,04 (0,63 – 1,73)	0,87
	**C**	35 (31)	56 (30)		
rs653752	**CC**	11 (19)	12 (13)	0,79 (0,30 – 2,06)	0,22
	**CG**	20 (35)	46 (49)	1,66 (0,80 – 3,44)	
	**GG**	26 (46)	36 (38)	1,0	
Allele	**G**	72 (63)	118 (63)	0,98 (0,61 – 1,59)	0,95
	**C**	42 (37)	70 (37)		

* chi-squared test; SNP-.single nucleotide polymorphism, *PGR*-progesterone receptor gene

Table S 12. Models of inheritance and their frequencies of six selected *PGR* SNPs in mothers with premature birth and the respective controls, when divided into two subgroups based on the mothers' BMI.

		Genotype [n (%)] **Mothers** BMI		Odds ratio (95% Confidence interval)	*P**
		Normal	Overweight		
rs10895068	**G/G**				
DOMINANT	**G/A-A/A**				
RECESSIVE	**G/G-G/A**				
	**A/A**				
OVERDOMINANT	**G/G-A/A**				
	**G/A**				
rs1042838	**G/G**	44 (77,2)	65 (69,2)	1	0,28
DOMINANT	**T/G-T/T**	13 (22,8)	29 (30,9)	1,51 (0,71-3,22)	
RECESSIVE	**G/G-T/G**	57 (100)	90 (95,7)	1	0,05
	**T/T**	0	4 (4,3)	-	
OVERDOMINANT	**G/G-T/T**	44 (77,2)	69 (73,4)	1	0,60
	**T/G**	13 (22,8)	25 (26,6)	1,23 (0,57-2,65)	
rs1042839	**C/C**	45 (79)	66 (70,2)	1	0,23
DOMINANT	**T/C-T/T**	12 (21,1)	28 (29,8)	1,59 (0,73-3,45)	
RECESSIVE	**C/C-T/C**	57 (100)	90 (95,7)	1	0,05
	**T/T**	0	4 (4,3)	-	
OVERDOMINANT	**C/C-T/T**	45 (79)	70 (74,5)	1	0,53
	**T/C**	12 (21,1)	24 (25,5)	1,29 (0,58-2,83)	
rs1942836	**T/T**	38 (66,7)	67 (71,3)	1	0,55
DOMINANT	**C/T-C/C**	19 (33,3)	27 (28,7)	0,81 (0,40-1,64)	
RECESSIVE	**T/T-C/T**	55 (96,5)	91 (96,8)	1	0,92
	**C/C**	2 (3,5)	3 (3,2)	0,91 (0,15-5,60)	
OVERDOMINANT	**T/T-C/C**	40 (70,2)	70 (74,5)	1	0,57
	**C/T**	17 (29,8)	24 (25,5)	0,81 (0,39-1,68)	
rs4754732	**T/T**	29 (50,9)	47 (50)	1	0,92
DOMINANT	**C/T-C/C**	28 (49,1)	47 (50)	1,04 (0,54-2,00)	
RECESSIVE	**T/T-C/T**	50 (87,7)	85 (90,4)	1	0,60
	**C/C**	7 (12,3)	9 (9,6)	0,76 (0,27-2,16)	
OVERDOMINANT	**T/T-C/C**	36 (63,2)	56 (59,6)	1	0,66
	**C/T**	21 (36,8)	38 (40,4)	1,16 (0,59-2,29)	
rs653752	**G/G**	26 (45,6)	36 (38,3)	1	0,38
DOMINANT	**C/G-C/C**	31 (54,4)	58 (61,7)	1,35 (0,69-2,63)	
RECESSIVE	**G/G-C/G**	46 (80,7)	82 (87,2)	1	0,28
	**C/C**	11 (19,3)	12 (12,8)	0,61 (0,25-1,50)	
OVERDOMINANT	**G/G-C/C**	37 (64,9)	48 (51,1)	1	0,10
	**C/G**	20 (35,1)	46 (48,9)	1,77 (0,90-3,49)	

* chi-squared test; SNP-.single nucleotide polymorphism, *PGR*-progesterone receptor gene

Table S 13 Genotype distribution and allele frequencies of six selected *PGR* SNPs in mothers with premature birth, when divided into two subgroups considering mothers' vaginal bleeding during pregnancy.

		**Mothers**			
		Genotype [n (%)] Vaginal bleeding during pregnancy		Odds ratio (95% Confidence interval)	*P**
		No	Yes		
rs10895068	**AA**	0	0	-	0,31
	**GA**	18 (16)	4 (10)	0,55 (0,18 – 1,74)	
	**GG**	92 (84)	37 (90)	1,0	
Allele	**G**	202 (92)	78 (95)	1,74 (0,57 – 5,30)	0,33
	**A**	18 (8)	4 (5)		
rs1042838	**GG**	81 (74)	28 (68)	1,0	0,42
	**TG**	27 (25)	11 (27)	1,18 (0,52 – 2,68)	
	**TT**	2 (2)	2 (5)	2,89 (0,39 – 21,52)	
Allele	**G**	189 (86)	67 (82)	0,73 (0,37 – 1,44)	0,37
	**T**	31 (14)	15 (18)		
rs1042839	**CC**	83 (75)	29 (71)	1,0	0,51
	**TC**	25 (23)	10 (24)	1,30 (0,57 – 2,99)	
	**TT**	2 (2)	2 (5)	2,96 (0,40 – 22,04)	
Allele	**C**	192 (87)	68 (83)	0,71 (0,35 – 1,42)	0,33
	**T**	28 (13)	14 (17)		
rs1942836	**CC**	5 (5)	0	-	0,31
	**CT**	32 (29)	9 (22)	0,64 (0,27 – 1,50)	
	**TT**	73 (66)	32 (78)	1,0	
Allele	**T**	178 (81)	73 (89)	1,91 (0,89 – 4,13)	0,09
	**C**	42 (19)	9 (11)		
rs4754732	**CC**	14 (13)	2 (5)	0,40 (0,08 – 1,92)	0,28
	**CT**	40 (36)	19 (46)	1,33 (0,63 – 2,81)	
	**TT**	56 (51)	20 (49)	1,0	
Allele	**T**	152 (69)	59 (72)	1,15 (0,66 – 2,01)	0,63
	**C**	68 (31)	23 (28)		
rs653752	**CC**	16 (15)	7 (17)	1,07 (0,38 – 3,04)	0,77
	**CG**	50 (45)	16 (39)	0,78 (0,36 – 1,72)	
	**GG**	44 (40)	18 (44)	1,0	
Allele	**G**	138 (63)	52 (63)	1,03 (0,61 – 1,74)	0,91
	**C**	82 (37)	30 (37)		

* chi-squared test; SNP-.single nucleotide polymorphism, *PGR*-progesterone receptor gene

Table S 14. Models of inheritance and their frequencies of six selected *PGR* SNPs in mothers with premature birth and the respective controls, when divided into two subgroups based on the mothers' vaginal bleeding during pregnancy.

		Genotype[n (%)] Mothers Bleeding during pregnancy		Odds ratio (95% Confidence interval)	*P**
		**No**	**Yes**		
rs10895068	**G/G**				
DOMINANT	**G/A-A/A**				
RECESSIVE	**G/G-G/A**				
	**A/A**				
OVERDOMINANT	**G/G-A/A**				
	**G/A**				
rs1042838	**G/G**	81 (73,6)	28 (68,3)	1	0,52
DOMINANT	**T/G-T/T**	29 (26,4)	13 (31,7)	1,30 (0,59-2,84)	
RECESSIVE	**G/G-T/G**	108 (98,2)	39 (95,1)	1	0,33
	**T/T**	2 (1,8)	2 (4,9)	2,77 (0,38-20,34)	
OVERDOMINANT	**G/G-T/T**	83 (75,5)	30 (73,2)	1	0,77
	**T/G**	27 (24,6)	11 (26,8)	1,13 (0,50-2,55)	
rs1042839	**C/C**	83 (75,5)	28 (68,3)	1	0,38
DOMINANT	**T/C-T/T**	27 (24,6)	13 (31,7)	1,43 (0,65-3,14)	
RECESSIVE	**C/C-T/C**	108 (98,2)	39 (95,1)	1	0,33
	**T/T**	2 (1,8)	2 (4,9)	2,77 (0,38-20,34)	
OVERDOMINANT	**C/C-T/T**	85 (77,3)	30 (73,2)	1	0,60
	**T/C**	25 (22,7)	11 (26,8)	1,25 (0,55-2,84)	
rs1942836	**T/T**	73 (66,4)	32 (78)	1	0,16
DOMINANT	**C/T-C/C**	37 (33,6)	9 (21,9)	0,55 (0,24-1,28)	
RECESSIVE	**T/T-C/T**	105 (95,5)	41 (100)	1	0,072
	**C/C**	5 (4,5)	0 (0)	-	
OVERDOMINANT	**T/T-C/C**	78 (70,9)	32 (78)	1	0,37
	**C/T**	32 (29,1)	9 (21,9)	0,69 (0,29-1,60)	
rs4754732	**T/T**	56 (50,9)	20 (48,8)	1	0,82
DOMINANT	**C/T-C/C**	54 (49,1)	21 (51,2)	1,09 (0,53-2,23)	
RECESSIVE	**T/T-C/T**	96 (87,3)	39 (95,1)	1	0,14
	**C/C**	14 (12,7)	2 (4,9)	0,35 (0,08-1,62)	
OVERDOMINANT	**T/T-C/C**	70 (63,6)	22 (53,7)	1	0,27
	**C/T**	40 (36,4)	19 (46,3)	1,51 (0,73-3,13)	
rs653752	**G/G**	44 (40)	18 (43,9)	1	0,67
DOMINANT	**C/G-C/C**	66 (60)	23 (56,1)	0,85 (0,41-1,76)	
RECESSIVE	**G/G-C/G**	94 (85,5)	34 (82,9)	1	0,70
	**C/C**	16 (14,6)	7 (17,1)	1,21 (0,46-3,19)	
OVERDOMINANT	**G/G-C/C**	60 (54,5)	25 (61)	1	0,48
	**C/G**	50 (45,5)	16 (39)	0,77 (0,37-1,60)	

* chi-squared test; SNP-.single nucleotide polymorphism, *PGR*-progesterone receptor gene

Table S 15. Genotype distribution and allele frequencies of six selected *PGR* SNPs in groups of term and preterm born newborns of mothers who had male newborns.

		Newborns			
		Genotype [n (%)] Male gender of newborns		Odds ratio (95% Confidence interval)	*P**
		Preterm birth (n = 86)	Term birth (n = 69)		
rs10895068	G/G	76 (88,4)	62 (89,9)	1	0,38
	G/A	10 (11,6)	6 (8,7)	0,74 (0,25-2,14)	
	A/A	0 (0)	1 (1,4)	-	
Alelle	G	162 (94)	130 (94)	1,0 (0,38 – 2,6)	0,99
	A	10 (6)	8 (6)		
rs1042838	G/G	64 (73,6)	51 (73,9)	1	0,93
	T/G	21 (24,1)	17 (24,6)	1,02 (0,49-2,12)	
	T/T	2 (2,3)	1 (1,4)	0,63 (0,06-7,12)	
Alelle	G	149 (86)	119 (86)	1,05 (0,55 – 1,9)	0,88
	T	25 (14)	19 (14)		
rs1042839	C/C	64 (73,6)	51 (73,9)	1	0,93
	T/C	21 (24,1)	17 (24,6)	1,02 (0,49-2,12)	
	T/T	2 (2,3)	1 (1,4)	0,63 (0,06-7,12)	
Alelle	C	64 (74)	51 (74)	0,98 (0,47 – 2,1)	0,97
	T	21 (24)	17 (25)		
rs1942836	T/T	60 (69)	56 (81,2)	1	0,20
	C/T	24 (27,6)	12 (17,4)	0,54 (0,24-1,17)	
	C/C	3 (3,5)	1 (1,4)	0,36 (0,04-3,53)	
Alelle	T	144 (83)	124 (90)	1,85 (0,94 – 3,6)	0,08
	C	30 (17)	14 (10)		
rs4754732	T/T	41 (47,7)	32 (46,4)	1	0,57
	C/T	32 (37,2)	30 (43,5)	1,20 (0,61-2,37)	
	C/C	13 (15,1)	7 (10,1)	0,69 (0,25-1,93)	
Alelle	T	114 (66)	94 (68)	1,09 (0,7 – 1,8)	0,73
	C	58 (34)	44 (32)		
rs653752	G/G	37 (42,5)	28 (40,6)	1	0,97
	C/G	37 (42,5)	30 (43,5)	1,07 (0,54-2,13)	
	C/C	13 (14,9)	11 (15,9)	1,12 (0,44-2,87)	
Alelle	G	111 (64)	86 (62)	0,94 (0,59-1,49)	0,79
	C	63 (36)	52 (38)		

* chi-squared test; SNP-.single nucleotide polymorphism, *PGR*-progesterone receptor gene

Table S 16. Genotype distribution and allele frequencies of six selected *PGR* SNPs between groups of preterm and term-born newborns of mothers older than 35 years.

		Newborns			
		Genotype (n (%)] 35 years and older mothers		Odds ratio (95% Confidence interval)	*P**
		Preterm birth (n = 51)	Term birth (n = 29)		
rs10895068	G/G	44 (86)	28 (97)	1	
	G/A	7 (14)	1 (3)	0,22 (0,03-1,8)	0,11
	A/A	0	0		
Allele	G	93 (93)	57 (98)	4,29 (0,5 – 35,8)	0,18
	A	7 (7)	1 (2)		
rs1042838	G/G	36 (70,6)	21 (72,4)	1	0,47
	T/G	14 (27,4)	6 (20,7)	0,73 (0,25-2,20)	
	T/T	1 (2)	2 (6,9)	3,43 (0,29-40,13)	
Allele	G	86 (84)	48 (83)	0,89 (0,38 – 2,1)	0,80
	T	16 (16)	10 (17)		
rs1042839	C/C	37 (72,5)	21 (72,4)	1	0,52
	T/C	13 (25,5)	6 (20,7)	0,81 (0,27-2,46)	
	T/T	1 (2)	2 (6,9)	3,52 (0,30-41,23)	
Allele	C	87 (85)	48 (83)	0,83 (0,35 – 1,9)	0,67
	T	15 (15)	10 (17)		
rs1942836	T/T	33 (64,7)	23 (79,3)	1	0,10
	C/T	17 (33,3)	4 (13,8)	0,34 (0,10-1,13)	
	C/C	1 (2)	2 (6,9)	2,87 (0,25-33,55)	
Allele	T	83 (81)	50 (86)	1,43 (0,58 – 3,5)	0,43
	C	19 (19)	8 (14)		
rs4754732	T/T	26 (52)	16 (55,2)	1	0,96
	C/T	18 (36)	10 (34,5)	0,90 (0,33-2,44)	
	C/C	6 (12)	3 (10,3)	0,81 (0,18-3,71)	
Allele	T	70 (70)	42 (72)	1,1 (0,55 – 2,31)	0,75
	C	30 (30)	16 (28)		
rs653752	G/G	21 (41,2)	10 (34,5)	1	0,83
	C/G	18 (35,3)	11 (37,9)	1,28 (0,44-3,71)	
	C/C	12 (23,5)	8 (27,6)	1,40 (0,43-4,51)	
Allele	G	60 (59)	31 (53)	0,80 (0,42 – 1,5)	0,51
	C	42 (41)	27 (47)		

* chi-squared test; SNP-.single nucleotide polymorphism, *PGR*-progesterone receptor gene

Table S 17. Genotype distribution and allele frequencies of six selected *PGR* SNPs between groups of preterm and term-born newborns of mothers who had vaginal bleeding during pregnancy.

		Newborns			
		Genotype (n (%)] Vaginal bleeding during pregnancy		Odds ratio (95% Confidence interval)	*P**
		Preterm birth (n = 42)	Term birth (n = 12)		
rs10895068	G/G	37 (90,2)	10 (83,3)	1	0,22
	G/A	4 (9,8)	1 (8,3)	0,93 (0,09-9,23)	
	A/A	0 (0)	1 (8,3)	-	
Alelle	G	78 (95)	21 (88)	0,36 (0,07 – 1,73)	0,20*
	A	4 (5)	3 (12)		
rs1042838	G/G	29 (69)	10 (83,3)	1	0,52
	T/G	12 (28,6)	2 (16,7)	0,48 (0,09-2,54)	
	T/T	1 (2,4)	0	-	
Alelle	G	70 (83)	22 (92)	2,2 (0,46 – 10,4)	0,32*
	T	14 (17)	2 (8)		
rs1042839	C/C	29 (69)	10 (83,3)	1	0,52
	T/C	12 (28,6)	2 (16,7)	0,48 (0,09-2,54)	
	T/T	1 (2,4)	0	-	
Alelle	C	70 (83)	22 (92)	2,2 (0,46 – 10,4)	0,32*
	T	14 (17)	2 (8)		
rs1942836	T/T	34 (81)	11 (91,7)	1	0,49
	C/T	6 (14,3)	1 (8,3)	0,52 (0,06-4,76)	
	C/C	2 (4,8)	0	-	
Alelle	T	74 (88)	23 (96)	3,1 (0,38 – 25,6)	0,29*
	C	10 (12)	1 (4)		
rs4754732	T/T	22 (53,7)	4 (33,3)	1	0,43
	C/T	13 (31,7)	6 (50)	2,54 (0,60-10,70)	
	C/C	6 (14,6)	2 (16,7)	1,83 (0,27-12,54)	
Alelle	T	57 (70)	14 (58)	0,61 (0,24 – 1,57)	0,31*
	C	25 (30)	10 (42)		
rs653752	G/G	18 (42,9)	5 (41,7)	1	>0,99
	C/G	17 (40,5)	5 (41,7)	1,06 (0,26-4,32)	
	C/C	7 (16,7)	2 (16,7)	1,03 (0,16-6,59)	
Alelle	G	53 (63)	15 (62)	0,97 (0,38 – 2,49)	0,96
	C	31 (37)	9 (38)		

* chi-squared test; SNP-.single nucleotide polymorphism, *PGR*-progesterone receptor gene
